# Crystal structure of bis­[(oxalato-κ^2^ *O* ^1^,*O* ^2^)(1,4,8,11-tetra­aza­cyclo­tetra­decane-κ^4^ *N*)chromium(III)] dichromate octa­hydrate from synchrotron X-ray data

**DOI:** 10.1107/S2056989017002614

**Published:** 2017-02-17

**Authors:** Dohyun Moon, Jong-Ha Choi

**Affiliations:** a Pohang Accelerator Laboratory, POSTECH, Pohang 37673, Republic of Korea; b Department of Chemistry, Andong National University, Andong 36729, Republic of Korea

**Keywords:** crystal structure, cyclam, chro­mium(III) complex, bidentate oxalato ligand, *cis*-V conformation, dichromate anion, hydrogen bonding, synchrotron radiation

## Abstract

The asymmetric unit of the title compound, [Cr(C_2_O_4_)(C_10_H_24_N_4_)]_2_[Cr_2_O_7_]·8H_2_O (C_10_H_24_N_4_ = 1,4,8,11-tetra­aza­cyclo­tetra­decane, cyclam; C_2_O_4_ = oxalate, ox) contains one [Cr(ox)(cyclam)]^+^ cation, one half of a dichromate anion that lies about an inversion centre so that the bridging O atom is equally disordered over two positions, and four water mol­ecules. The terminal O atoms of the dichromate anion are also disordered over two positions with a refined occupancy ratio 0.586 (6):0.414 (6). The Cr^III^ ion is coordinated by the four N atoms of the cyclam ligand and one bidentate oxalato ligand in a *cis* arrangement, resulting in a distorted octa­hedral geometry. The Cr—N(cyclam) bond lengths are in the range 2.069 (2)–2.086 (2) Å, while the average Cr—O(ox) bond length is 1.936 Å. The macrocyclic cyclam moiety adopts the *cis*-V conformation. The dichromate anion has a staggered conformation. The crystal structure is stabilized by inter­molecular hydrogen bonds involving the cyclam N—H groups and water O—H groups as donors, and the O atoms of oxalate ligand, water mol­ecules and the Cr_2_O_7_^2−^ anion as acceptors, giving rise to a three-dimensional network.

## Abstract 2

The asymmetric unit of the title compound comprises of one complex cation, one half of a [Cr_2_O_7_]^2−^ anion and four water mol­ecules. The Cr^III^ ion has a distorted octa­hedral coordination by four N atoms of the cyclam ligand and one bidentate oxalate ligand in the *cis* positions; the conformation of the dichromate anion is staggered.

## Chemical context   

Chromium (Cr) is considered a trace element essential for the proper functioning of living organisms and is also a highly toxic material (Yusof & Malek, 2009 ▸). Cr can exist in all oxidation states from 0 to VI, the most common oxidation states in water being Cr^III^ and Cr^VI^. In an aqueous environment, the toxicity of Cr^VI^ has been shown to be greater than that of Cr^III^ (Guzel *et al.*, 2016 ▸). Transition metal complexes of the cyclam (1,4,8,11-tetra­aza­cyclo­tetra­decane, C_10_H_24_N_4_) ligand have been the subject of numerous investigations because of their particular conformational stereochemistry (Poon & Pun, 1980 ▸; Choi, 2009 ▸; Subhan *et al.*, 2011 ▸). Recently, it has been found that cyclam derivatives and their metal complexes exhibit anti-HIV activity (Ronconi & Sadler, 2007 ▸; De Clercq, 2010 ▸; Ross *et al.*, 2012 ▸). The conformation of the macrocyclic ligand is a very important factor for co-receptor recognition. Therefore, knowledge of the conformation and hydrogen-bonding inter­actions in Cr^III^–Cr^VI^ complex systems containing the cyclam ligand has become important in the development of new anti*-*HIV drugs (De Clercq, 2010 ▸). The use of such complexes for the more effective removal of toxic metals is also important (Guzel *et al.*, 2016 ▸). As part of a study of the conformation and structure of (cyclam)chromium(III) complexes with auxiliary ligand(s) and various anions, we report here the structural characterization of the new complex salt, [Cr(C_2_O_4_)(C_10_H_24_N_4_)]_2_[Cr_2_O_7_]·8H_2_O, (I).
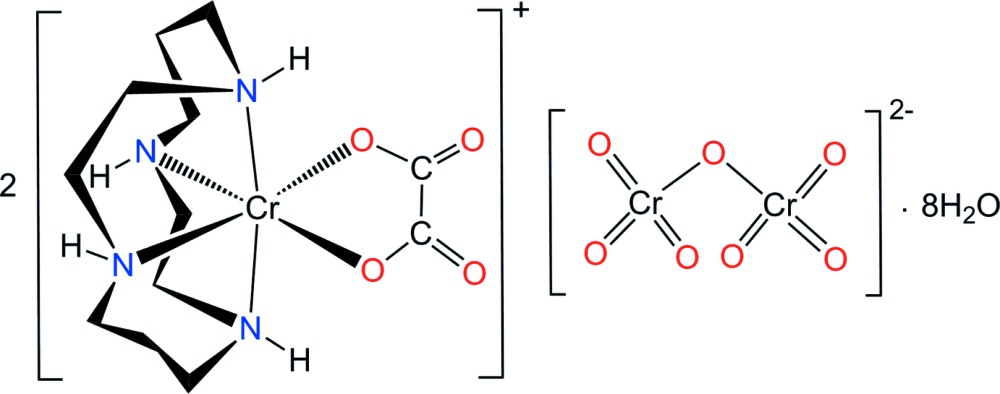


## Structural commentary   

**Figure 1:**
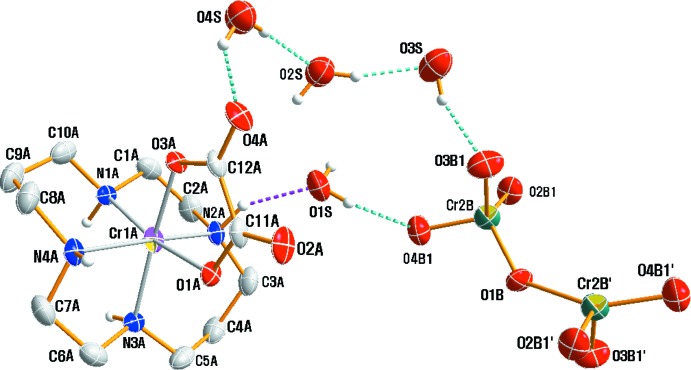
A perspective view of the asymmetric unit of the title of compound, (I), with the dichromate anion, which lies about an inversion centre, drawn in full. Displacement ellipsoids are drawn at the 30% probability level and primed atoms are related by the symmetry operation (2 − *x*, −*y*, 1 − *z*). For clarity, only the major disorder components are shown for the disordered dichromate anion.

An ellipsoid plot of the mol­ecular components in (I) is shown in Fig. 1 ▸ along with the atom-numbering scheme. The structure is another example of a [Cr(ox)(cyclam)]^+^ cation (Choi *et al.*, 2004*b* ▸; Moon & Choi, 2016*b* ▸), but with a different counter-anion. The asymmetric unit contains one [Cr(ox)(cyclam)]^+^ cation, one half of a Cr_2_O_7_^2−^ anion (completed by inversion symmetry with the bridging O atom disordered about the inversion centre) and four non-coordinating water mol­ecules. The three terminal O atoms of the dichromate anion are also disordered over two positions with occupancy ratio of the atom pairs O2*B*1/O2*B*2, O3*B*1/O3*B*2 and O4*B*1/O4*B*2 converging at 0.586 (6):0.414 (6). The conformation of the cyclam ligand can be described as *cis*-V (*anti*–*anti*) (Subhan *et al.*, 2011 ▸). In the complex cation, the Cr^III^ ion is coordinated by the four nitro­gen atoms of the cyclam ligand in a folded conformation. Two oxygen atoms of the oxalato ligand complete the distorted octa­hedral coordination sphere. The Cr—N bond lengths from the donor atoms of cyclam ligand lie in the range 2.069 (2) to 2.086 (2) Å, in good agreement with those determined in *cis-*[Cr(N_3_)_2_(cyclam)]ClO_4_ [2.069 (3)–2.103 (3) Å] (Meyer *et al.*, 1998 ▸), *cis*-[Cr(ONO)_2_(cyclam)]NO_2_ [2.0874 (16)–2.0916 (15) Å] (Choi *et al.*, 2004*a* ▸), [Cr(acac)(cyclam)](ClO_4_)_2_·0.5H_2_O [2.070 (5)–2.089 (5) Å] (acac = acetyl­acetonate; Subhan *et al.*, 2011 ▸), *cis*-[Cr(NCS)_2_(cyclam)]NCS [2.0851 (14)–2.0897 (14) Å] (Moon *et al.*, 2013 ▸) and [CrCl_2_(cyclam)][Cr(ox)(cyclam)](ClO_4_)_2_ [2.075 (5)–2.096 (5) Å] (Moon & Choi, 2016*b* ▸). However, the Cr—N bond lengths of the cyclam ligand with a *cis* geometry are slightly longer than those found in *trans-*[Cr(NCS)_2_(cyclam)]ClO_4_ [2.046 (2)–2.060 (2) Å] (Friesen *et al.*, 1997 ▸), *trans*-[Cr(ONO)_2_)(cyclam)]BF_4_ [2.064 (4)–2.073 (4) Å] (De Leo *et al.*, 2000 ▸), *trans-*[Cr(NH_3_)_2_(cyclam)][ZnCl_4_]Cl·H_2_O [2.0501 (15)–2.0615 (15) Å] (Moon & Choi, 2016*a* ▸) and *trans*-[Cr(nic-O)_2_(cyclam)]ClO_4_ [2.058 (4)–2.064 (4) Å] (nic-O = O-coordinated nicotinate; Choi, 2009 ▸).

The Cr1*A*—O1*A* distance [1.9665 (16) Å] in the oxalate ligand is very slightly longer than the Cr1*A*—O3*A* [1.9600 (16) Å] bond length. This elongation may be attributed to the weak hydrogen bond formed by O1*A* (*x*, −*y* + 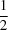, *z* − 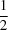) with the O3*S*—H2*O*3 atoms of a water mol­ecule. The mean Cr—O bond length is comparable to the mean values of 1.959, 1.956 and 1.969 Å observed in [Cr(ox)(cyclam)]ClO_4_ (Choi *et al.*, 2004*b* ▸), [CrCl_2_(cyclam)][Cr(ox)(cyclam)](ClO_4_)_2_ (Moon & Choi, 2016*b* ▸) and K_3_[Cr(ox)_3_]·3H_2_O (Taylor, 1978 ▸), respectively. The five- and six-membered chelate rings of the cyclam ligand adopt *gauche* and stable chair conformations, respectively. As expected for a bidentate ox ligand, the O1*A*—Cr1*A*—O3*A* bite angle 82.34 (7)° is considerably less than 90°, while the folding angle of the cyclam in the [Cr(ox)(cyclam)]^+^ cation is 98.97 (8)°. The significant distortion of the octa­hedron and the larger folding angle in the [Cr(ox)(cyclam)]^+^ cation seem to arise from the small bite angle of the bidentate oxalato ligand.

It is of inter­est to compare the conformation of the Cr_2_O_7_^2−^ anion with that found in other ionic crystals. In (I), the Cr_2_O_7_^2−^ anion exhibits a staggered conformation whereas a nearly eclipsed conformation is observed for (C_9_H_14_N)_2_[Cr_2_O_7_] and (C_10_H_22_N_2_)[Cr_2_O_7_], when viewed along the backbone of the dichromate anion (Trabelsi *et al.*, 2015 ▸; Chebbi *et al.*, 2016 ▸). This structural conformation of dichromate seems to depend on the size of the associated counter-cation (Moon *et al.*, 2015 ▸, 2017 ▸). The O1*B*—Cr2*B*—O bond angles range from 107.1 (3) to 117.0 (3)°; while the terminal Cr2*B*—O bond lengths vary from 1.572 (12) to 1.673 (5) Å, with a mean terminal Cr2*B*—O bond length of 1.627 Å. The bridging Cr2*B*—O1*B* bond is 1.684 (4) Å long, with the Cr2*B*—O1*B*—Cr2*B*(−*x* + 2, −*y*, −*z* + 1) bond angle of 136.0 (3)°. These values are similar to those reported for the anions in the structures of [Cr(urea)_6_][Cr_2_O_7_]Br·H_2_O (Moon *et al.*, 2015 ▸) and [Cr(NCS)_2_(cyclam)]_2_[Cr_2_O_7_]·H_2_O (Moon *et al.*, 2017 ▸). A further distortion of the anion undoubtedly results from its involvement in hydrogen-bonding inter­actions with the solvent water mol­ecules (see *Supra­molecular features*).

## Supra­molecular features   

**Figure 2:**
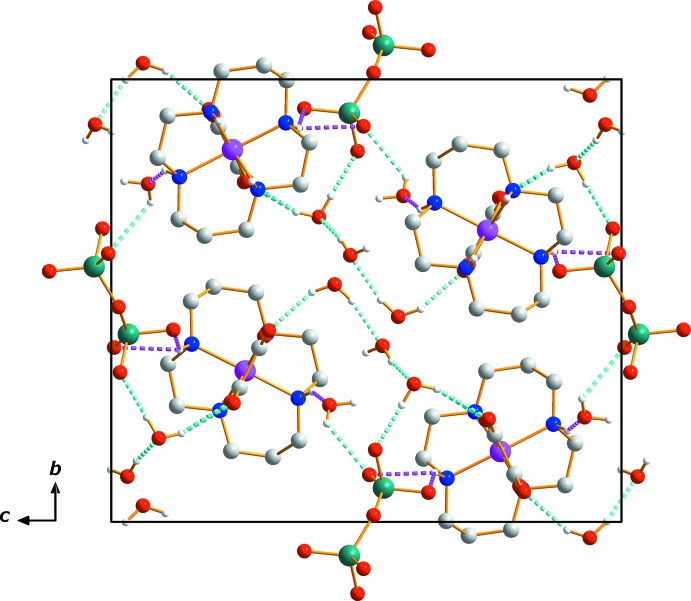
The crystal packing in compound (I), viewed perpendicular to the *bc* plane. Dashed lines represent N—H⋯O (pink) and O—H⋯O (cyan) hydrogen-bonding inter­actions, respectively. C-bound H atoms have been omitted.

In the asymmetric unit, O—H⋯O and N—H⋯O hydrogen bonds link the water mol­ecules to the Cr_2_O_7_^2−^ anion, [Cr(ox)(cyclam)]^+^ cation and other water mol­ecules, while N—H⋯O hydrogen bonds involving the cyclam N–H groups and the O atoms of oxalate inter­connect two [Cr(ox)(cyclam)]^+^ cations (Table 1 ▸, Figs. 1 ▸ and 2 ▸). An extensive array of these contacts generate a three-dimensional network of mol­ecules (Fig. 2 ▸), and these hydrogen-bonding inter­actions help to stabilize the crystal structure.

## Database survey   

A search of the Cambridge Structural Database (Version 5.37, Feb 2016 with two updates; Groom *et al.*, 2016 ▸) gave just one hit for a [Cr(C_2_O_4_)(C_10_H_24_N_4_)]^+^ unit, namely the complex [Cr(ox)(cyclam)]ClO_4_ (Choi *et al.*, 2004*b* ▸). However, the structure of [CrCl_2_(cyclam)][Cr(ox)(cyclam)](ClO_4_)_2_ (Moon & Choi, 2016*b* ▸) has also been reported recently. Until now, no structure of the [Cr(ox)(cyclam)]^+^ cation with a dichromate counter-anion has been deposited.

## Synthesis and crystallization   

The free ligand cyclam (98%) was purchased from Sigma–Aldrich and used without further purification. All chemicals were reagent grade materials, and were used as received. The starting material, [Cr(ox)(cyclam)]ClO_4_ was prepared according to the literature method (House & McKee, 1984 ▸). The perchlorate salt of the complex (0.03 g) was dissolved in 10 mL of distilled water at 347 K. The solution was filtered and the filtrate was added to 5 mL of water containing solid K_2_Cr_2_O_7_ (0.02 g). Orange block-like crystals of (I) suitable for X-ray structural analysis were obtained after one week of slow evaporation at room temperature.

## Refinement   

Crystal data, data collection and structure refinement details are summarized in Table 2 ▸. All H atoms were placed in geometrically idealized positions and constrained to ride on their parent atoms, with C—H = 0.97 Å and N—H = 0.98 Å, and with *U*_iso_(H) values of 1.2*U*_eq_ of the parent atoms. The hydrogen atoms of the solvent water mol­ecules were assigned based on a difference-Fourier map, and the O—H distance and the H—O—H angle were restrained using DFIX and DANG constraints. The terminal O atoms of the dichromate anion are positionally disordered over two sets of sites. The occupancies of the respective pairs, O2*B*1/O2*B*2, O3*B*1/O3*B*2 and O4*B*1/O4*B*2, were refined freely and, for the O2*B*2 and O3*B*2 atoms, ISOR restraints were applied. The occupancy ratio refined to 0.586 (6):0.414 (6). The bridging O1*B* atom of the dichromate anion is also disordered, in this case about the inversion centre. Consequently the components were refined at half-occupancy. The bridging atoms O1*B*/O1*B* (−*x* + 2, −*y*, −*z* + 1) sites were refined using EXYZ/EADP restraints.

## Supplementary Material

Crystal structure: contains datablock(s) I. DOI: 10.1107/S2056989017002614/sj5521sup1.cif

Structure factors: contains datablock(s) I. DOI: 10.1107/S2056989017002614/sj5521Isup2.hkl

CCDC reference: 1532785

Additional supporting information:  crystallographic information; 3D view; checkCIF report

## Figures and Tables

**Table 1:** Hydrogen-bond geometry (Å, °)

*D*—H⋯*A*	*D*—H	H⋯*A*	*D*⋯*A*	*D*—H⋯*A*
N1*A*—H1*A*⋯O4*A* ^i^	0.98	1.99	2.804 (3)	139
N2*A*—H2*A*⋯O1*S*	0.98	2.00	2.894 (3)	150
N3*A*—H3*A*⋯O2*A* ^i^	0.98	1.89	2.842 (3)	163
N4*A*—H4*A*⋯O2*B*1^ii^	0.98	2.25	3.100 (16)	144
N4*A*—H4*A*⋯O4*B*1^ii^	0.98	2.37	3.108 (6)	132
N4*A*—H4*A*⋯O4*B*2^ii^	0.98	2.15	3.05 (2)	151
O1*S*—H1*O*1⋯O4*B*1	0.85 (1)	2.33 (5)	2.876 (6)	123 (5)
O1*S*—H1*O*1⋯O3*B*2	0.85 (1)	2.20 (4)	2.903 (10)	141 (5)
O2*S*—H2*O*2⋯O3*S*	0.85 (1)	1.91 (2)	2.729 (6)	164 (6)
O3*S*—H1*O*3⋯O3*B*1	0.85 (1)	1.93 (3)	2.725 (6)	156 (7)
O3*S*—H1*O*3⋯O3*B*2	0.85 (1)	2.28 (2)	3.113 (10)	167 (6)
O3*S*—H2*O*3⋯O1*A* ^iii^	0.85 (1)	2.52 (3)	3.301 (4)	154 (6)
O3*S*—H2*O*3⋯O2*A* ^iii^	0.85 (1)	2.21 (5)	2.911 (5)	140 (6)
O4*S*—H1*O*4⋯O4*A*	0.84 (1)	2.11 (4)	2.834 (4)	144 (6)
O4*S*—H2*O*4⋯O2*S*	0.85 (1)	1.89 (2)	2.723 (5)	166 (6)

Symmetry codes: (i) 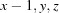; (ii) 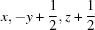; (iii) 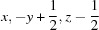.

**Table 2:** Experimental details

Crystal data	
Chemical formula	[Cr(C_2_O_4_)(C_10_H_24_N_4_)]_2_[Cr_2_O_7_]·8H_2_O
*M*_r_	1040.83
Crystal system, space group	Monoclinic, *P*2_1_/*c*
Temperature (K)	298
*a*, *b*, *c* (Å)	7.8270 (16), 15.407 (3), 18.086 (4)
β (°)	100.86 (3)
*V* (Å^3^)	2141.9 (8)
*Z*	2
Radiation type	Synchrotron, λ = 0.610 Å
μ (mm^−1^)	0.71
Crystal size (mm)	0.15 × 0.09 × 0.08

Data collection	
Diffractometer	ADSC Q210 CCD area detector
Absorption correction	Empirical (using intensity measurements) (*HKL3000sm *SCALEPACK**; Otwinowski & Minor, 1997 ▸)
*T*_min_, *T*_max_	0.889, 1.000
No. of measured, independent and observed [*I* > 2σ(*I*)] reflections	21270, 5775, 4844
*R*_int_	0.027
(sin θ/λ)_max_ (Å^−1^)	0.693

Refinement	
*R*[*F* ^2^ > 2σ(*F* ^2^)], *wR*(*F* ^2^), *S*	0.048, 0.145, 1.04
No. of reflections	5775
No. of parameters	324
No. of restraints	24
H-atom treatment	H atoms treated by a mixture of independent and constrained refinement
Δρ_max_, Δρ_min_ (e Å^−3^)	1.62, −0.69

Computer programs: *PAL BL2D-SMDC* (Shin *et al.*, 2016 ▸), *HKL3000sm* (Otwinowski & Minor, 1997 ▸), *SHELXT2014* (Sheldrick, 2015*a* ▸), *SHELXL2016* (Sheldrick, 2015*b* ▸), *DIAMOND 4* (Putz & Brandenburg, 2014 ▸) and *publCIF* (Westrip, 2010 ▸).

## supplementary crystallographic information

### Crystal data

**Table:**

[Cr(C_2_O_4_)(C_10_H_24_N_4_)]_2_[Cr_2_O_7_]·8H_2_O	*F*(000) = 1088
*M**_r_* = 1040.83	*D*_x_ = 1.614 Mg m^−^^3^
Monoclinic, *P*2_1_/*c*	Synchrotron radiation, λ = 0.610 Å
*a* = 7.8270 (16) Å	Cell parameters from 63673 reflections
*b* = 15.407 (3) Å	θ = 0.4–33.7°
*c* = 18.086 (4) Å	µ = 0.71 mm^−^^1^
β = 100.86 (3)°	*T* = 298 K
*V* = 2141.9 (8) Å^3^	Block, orange
*Z* = 2	0.15 × 0.09 × 0.08 mm

### Data collection

**Table:**

ADSC Q210 CCD area detector diffractometer	4844 reflections with *I* > 2σ(*I*)
Radiation source: PLSII 2D bending magnet	*R*_int_ = 0.027
ω scan	θ_max_ = 25.0°, θ_min_ = 2.3°
Absorption correction: empirical (using intensity measurements) (*HKL3000sm SCALEPACK*; Otwinowski & Minor, 1997)	*h* = −10→10
*T*_min_ = 0.889, *T*_max_ = 1.000	*k* = −20→20
21270 measured reflections	*l* = −25→25
5775 independent reflections	

### Refinement

**Table:**

Refinement on *F*^2^	Hydrogen site location: mixed
Least-squares matrix: full	H atoms treated by a mixture of independent and constrained refinement
*R*[*F*^2^ > 2σ(*F*^2^)] = 0.048	*w* = 1/[σ^2^(*F*_o_^2^) + (0.0902*P*)^2^ + 1.1358*P*] where *P* = (*F*_o_^2^ + 2*F*_c_^2^)/3
*wR*(*F*^2^) = 0.145	(Δ/σ)_max_ = 0.002
*S* = 1.04	Δρ_max_ = 1.62 e Å^−^^3^
5775 reflections	Δρ_min_ = −0.69 e Å^−^^3^
324 parameters	Extinction correction: SHELXL2016 (Sheldrick, 2015*b*), Fc^*^=kFc[1+0.001xFc^2^λ^3^/sin(2θ)]^-1/4^
24 restraints	Extinction coefficient: 0.037 (3)

### Special details

**Table:**

Geometry. All esds (except the esd in the dihedral angle between two l.s. planes) are estimated using the full covariance matrix. The cell esds are taken into account individually in the estimation of esds in distances, angles and torsion angles; correlations between esds in cell parameters are only used when they are defined by crystal symmetry. An approximate (isotropic) treatment of cell esds is used for estimating esds involving l.s. planes.

### Fractional atomic coordinates and isotropic or equivalent isotropic displacement parameters (Å^2^)

**Table:**

	*x*	*y*	*z*	*U*_iso_*/*U*_eq_	Occ. (<1)
Cr1A	0.38205 (4)	0.34112 (2)	0.73752 (2)	0.02641 (13)	
O1A	0.59297 (19)	0.27010 (12)	0.76591 (10)	0.0396 (4)	
O2A	0.8739 (2)	0.26797 (16)	0.76043 (14)	0.0626 (6)	
O3A	0.54391 (19)	0.41929 (11)	0.69995 (10)	0.0396 (4)	
O4A	0.8222 (2)	0.43225 (16)	0.69140 (14)	0.0598 (6)	
N1A	0.1816 (2)	0.42579 (13)	0.69489 (12)	0.0378 (4)	
H1A	0.075547	0.405210	0.710638	0.045*	
N2A	0.3113 (2)	0.27935 (15)	0.63360 (12)	0.0409 (4)	
H2A	0.403674	0.293806	0.605848	0.049*	
N3A	0.2422 (2)	0.25158 (13)	0.78721 (12)	0.0390 (4)	
H3A	0.119637	0.269026	0.775430	0.047*	
N4A	0.4158 (3)	0.40170 (14)	0.84214 (11)	0.0406 (4)	
H4A	0.535961	0.389115	0.866550	0.049*	
C1A	0.1568 (4)	0.4172 (2)	0.61046 (18)	0.0582 (8)	
H1AA	0.049076	0.445014	0.586857	0.070*	
H1AB	0.252088	0.445169	0.592350	0.070*	
C2A	0.1514 (4)	0.3226 (2)	0.59104 (18)	0.0612 (8)	
H2AA	0.048592	0.296162	0.604142	0.073*	
H2AB	0.145782	0.315414	0.537358	0.073*	
C3A	0.3008 (4)	0.1838 (2)	0.6321 (2)	0.0609 (8)	
H3AA	0.417078	0.160207	0.647097	0.073*	
H3AB	0.256590	0.165122	0.580818	0.073*	
C4A	0.1857 (4)	0.1466 (2)	0.6829 (2)	0.0638 (9)	
H4AA	0.071236	0.172674	0.669584	0.077*	
H4AB	0.172624	0.084736	0.673435	0.077*	
C5A	0.2513 (4)	0.16009 (18)	0.7649 (2)	0.0586 (8)	
H5AA	0.183071	0.125105	0.793222	0.070*	
H5AB	0.371013	0.140530	0.777515	0.070*	
C6A	0.3031 (4)	0.2623 (2)	0.87113 (18)	0.0597 (8)	
H6AA	0.224823	0.232351	0.898189	0.072*	
H6AB	0.418710	0.238033	0.886411	0.072*	
C7A	0.3053 (5)	0.3571 (3)	0.88827 (18)	0.0640 (8)	
H7AA	0.187953	0.380198	0.876854	0.077*	
H7AB	0.351179	0.366653	0.941312	0.077*	
C8A	0.3993 (4)	0.4980 (2)	0.8437 (2)	0.0605 (8)	
H8AA	0.493986	0.523712	0.823521	0.073*	
H8AB	0.410781	0.516864	0.895598	0.073*	
C9A	0.2285 (4)	0.5313 (2)	0.7993 (2)	0.0657 (9)	
H9AA	0.133959	0.502373	0.817229	0.079*	
H9AB	0.219591	0.592861	0.809118	0.079*	
C10A	0.2063 (4)	0.51784 (19)	0.7163 (2)	0.0595 (8)	
H10A	0.106358	0.550800	0.691276	0.071*	
H10B	0.308132	0.539863	0.699087	0.071*	
C11A	0.7305 (3)	0.30305 (18)	0.74841 (14)	0.0415 (5)	
C12A	0.7022 (3)	0.39281 (18)	0.70995 (14)	0.0412 (5)	
Cr2B	0.86158 (6)	0.07641 (3)	0.46646 (2)	0.04561 (15)	
O1B	0.9327 (6)	−0.0145 (4)	0.5141 (3)	0.0780 (16)	0.5
O2B1	0.814 (2)	0.0661 (9)	0.3785 (7)	0.068 (3)	0.586 (6)
O3B1	0.9854 (7)	0.1624 (3)	0.4827 (3)	0.0797 (17)	0.586 (6)
O4B1	0.6779 (6)	0.1061 (4)	0.4942 (3)	0.0811 (16)	0.586 (6)
O2B2	0.7152 (9)	0.0097 (6)	0.4864 (4)	0.093 (3)	0.414 (6)
O3B2	0.8779 (13)	0.1556 (6)	0.5165 (5)	0.097 (3)	0.414 (6)
O4B2	0.812 (3)	0.0895 (14)	0.3789 (11)	0.075 (5)	0.414 (6)
O1S	0.6259 (5)	0.2636 (2)	0.57243 (19)	0.0909 (10)	
H1O1	0.698 (7)	0.222 (3)	0.577 (3)	0.136*	
H2O1	0.596 (8)	0.269 (4)	0.5252 (8)	0.136*	
O2S	0.6340 (5)	0.3977 (2)	0.4684 (2)	0.0935 (9)	
H1O2	0.576 (7)	0.382 (4)	0.501 (3)	0.140*	
H2O2	0.698 (7)	0.362 (3)	0.450 (3)	0.140*	
O3S	0.8777 (6)	0.3113 (2)	0.4068 (2)	0.1014 (11)	
H1O3	0.880 (9)	0.264 (2)	0.431 (3)	0.152*	
H2O3	0.827 (8)	0.300 (4)	0.3622 (14)	0.152*	
O4S	0.7101 (5)	0.5364 (2)	0.56219 (18)	0.0920 (9)	
H1O4	0.708 (9)	0.519 (3)	0.6063 (12)	0.138*	
H2O4	0.675 (8)	0.499 (3)	0.528 (2)	0.138*	

### Atomic displacement parameters (Å^2^)

**Table:**

	*U*^11^	*U*^22^	*U*^33^	*U*^12^	*U*^13^	*U*^23^
Cr1A	0.01035 (16)	0.0353 (2)	0.03406 (19)	0.00068 (10)	0.00548 (11)	−0.00334 (12)
O1A	0.0188 (7)	0.0472 (10)	0.0521 (9)	0.0076 (6)	0.0050 (6)	−0.0005 (7)
O2A	0.0197 (8)	0.0833 (16)	0.0835 (15)	0.0165 (8)	0.0066 (8)	−0.0129 (12)
O3A	0.0177 (7)	0.0490 (10)	0.0546 (10)	−0.0027 (6)	0.0133 (6)	0.0023 (7)
O4A	0.0229 (8)	0.0871 (16)	0.0737 (14)	−0.0124 (9)	0.0203 (8)	−0.0010 (11)
N1A	0.0173 (8)	0.0424 (11)	0.0544 (11)	0.0062 (7)	0.0089 (7)	0.0063 (8)
N2A	0.0258 (9)	0.0561 (12)	0.0409 (10)	−0.0004 (8)	0.0066 (7)	−0.0124 (9)
N3A	0.0216 (8)	0.0435 (11)	0.0537 (11)	−0.0007 (7)	0.0115 (8)	0.0059 (8)
N4A	0.0273 (9)	0.0533 (12)	0.0408 (10)	−0.0009 (8)	0.0058 (7)	−0.0127 (8)
C1A	0.0373 (13)	0.080 (2)	0.0565 (16)	0.0114 (13)	0.0069 (11)	0.0225 (15)
C2A	0.0434 (15)	0.087 (2)	0.0473 (15)	0.0015 (15)	−0.0069 (12)	−0.0098 (14)
C3A	0.0495 (16)	0.0600 (18)	0.073 (2)	−0.0032 (13)	0.0114 (14)	−0.0286 (15)
C4A	0.0488 (17)	0.0501 (17)	0.093 (2)	−0.0108 (12)	0.0130 (16)	−0.0183 (15)
C5A	0.0442 (15)	0.0407 (15)	0.092 (2)	−0.0025 (10)	0.0169 (15)	0.0052 (13)
C6A	0.0497 (16)	0.077 (2)	0.0546 (16)	0.0013 (14)	0.0162 (13)	0.0168 (14)
C7A	0.067 (2)	0.086 (2)	0.0446 (15)	−0.0042 (17)	0.0243 (14)	−0.0113 (14)
C8A	0.0517 (17)	0.0561 (18)	0.074 (2)	−0.0037 (13)	0.0125 (14)	−0.0275 (15)
C9A	0.0498 (17)	0.0528 (18)	0.097 (3)	0.0099 (13)	0.0195 (16)	−0.0179 (16)
C10A	0.0376 (14)	0.0447 (15)	0.098 (2)	0.0084 (11)	0.0189 (14)	0.0074 (14)
C11A	0.0160 (9)	0.0608 (15)	0.0470 (12)	0.0055 (9)	0.0041 (8)	−0.0140 (10)
C12A	0.0177 (9)	0.0612 (15)	0.0472 (12)	−0.0071 (9)	0.0120 (8)	−0.0119 (10)
Cr2B	0.0406 (2)	0.0553 (3)	0.0379 (2)	0.00143 (17)	−0.00027 (16)	0.00325 (16)
O1B	0.058 (3)	0.083 (3)	0.094 (4)	0.032 (3)	0.016 (3)	0.051 (3)
O2B1	0.074 (5)	0.082 (6)	0.042 (4)	0.010 (4)	−0.002 (3)	−0.009 (4)
O3B1	0.076 (3)	0.065 (3)	0.086 (3)	−0.025 (2)	−0.016 (3)	−0.006 (2)
O4B1	0.066 (3)	0.095 (4)	0.087 (3)	0.011 (2)	0.027 (2)	−0.005 (3)
O2B2	0.065 (4)	0.126 (6)	0.094 (5)	−0.007 (4)	0.027 (3)	0.045 (4)
O3B2	0.096 (3)	0.095 (3)	0.097 (3)	0.0059 (19)	0.0135 (19)	−0.0124 (18)
O4B2	0.050 (5)	0.113 (13)	0.060 (7)	0.023 (7)	0.009 (5)	0.047 (7)
O1S	0.097 (2)	0.102 (2)	0.089 (2)	0.0078 (17)	0.0558 (19)	−0.0074 (17)
O2S	0.110 (3)	0.082 (2)	0.089 (2)	0.0060 (18)	0.0202 (18)	−0.0030 (16)
O3S	0.131 (3)	0.084 (2)	0.082 (2)	0.013 (2)	0.003 (2)	−0.0045 (16)
O4S	0.111 (3)	0.091 (2)	0.0739 (18)	0.0025 (19)	0.0187 (19)	−0.0037 (15)

### Geometric parameters (Å, º)

**Table:**

Cr1A—O3A	1.9600 (16)	C5A—H5AB	0.9700
Cr1A—O1A	1.9665 (16)	C6A—C7A	1.493 (5)
Cr1A—N3A	2.069 (2)	C6A—H6AA	0.9700
Cr1A—N1A	2.0739 (19)	C6A—H6AB	0.9700
Cr1A—N4A	2.081 (2)	C7A—H7AA	0.9700
Cr1A—N2A	2.086 (2)	C7A—H7AB	0.9700
O1A—C11A	1.283 (3)	C8A—C9A	1.513 (5)
O2A—C11A	1.228 (3)	C8A—H8AA	0.9700
O3A—C12A	1.284 (3)	C8A—H8AB	0.9700
O4A—C12A	1.218 (3)	C9A—C10A	1.493 (5)
N1A—C10A	1.473 (4)	C9A—H9AA	0.9700
N1A—C1A	1.508 (4)	C9A—H9AB	0.9700
N1A—H1A	0.9800	C10A—H10A	0.9700
N2A—C3A	1.475 (4)	C10A—H10B	0.9700
N2A—C2A	1.497 (4)	C11A—C12A	1.545 (4)
N2A—H2A	0.9800	Cr2B—O3B2	1.511 (8)
N3A—C5A	1.472 (4)	Cr2B—O4B2	1.571 (19)
N3A—C6A	1.512 (4)	Cr2B—O2B1	1.572 (12)
N3A—H3A	0.9800	Cr2B—O2B2	1.630 (7)
N4A—C7A	1.479 (4)	Cr2B—O3B1	1.635 (4)
N4A—C8A	1.491 (4)	Cr2B—O4B1	1.673 (5)
N4A—H4A	0.9800	Cr2B—O1B	1.684 (4)
C1A—C2A	1.498 (5)	Cr2B—O1B^i^	1.847 (4)
C1A—H1AA	0.9700	O1B—O1B^i^	1.332 (10)
C1A—H1AB	0.9700	O1B—O2B2	1.723 (9)
C2A—H2AA	0.9700	O1S—H1O1	0.845 (10)
C2A—H2AB	0.9700	O1S—H2O1	0.847 (10)
C3A—C4A	1.516 (5)	O2S—H1O2	0.841 (10)
C3A—H3AA	0.9700	O2S—H2O2	0.845 (10)
C3A—H3AB	0.9700	O3S—H1O3	0.845 (10)
C4A—C5A	1.489 (5)	O3S—H2O3	0.848 (10)
C4A—H4AA	0.9700	O4S—H1O4	0.843 (10)
C4A—H4AB	0.9700	O4S—H2O4	0.851 (10)
C5A—H5AA	0.9700		
			
O3A—Cr1A—O1A	82.34 (7)	N3A—C5A—H5AB	109.1
O3A—Cr1A—N3A	171.83 (8)	C4A—C5A—H5AB	109.1
O1A—Cr1A—N3A	90.14 (8)	H5AA—C5A—H5AB	107.8
O3A—Cr1A—N1A	88.72 (7)	C7A—C6A—N3A	107.7 (2)
O1A—Cr1A—N1A	170.42 (8)	C7A—C6A—H6AA	110.2
N3A—Cr1A—N1A	98.97 (8)	N3A—C6A—H6AA	110.2
O3A—Cr1A—N4A	93.44 (8)	C7A—C6A—H6AB	110.2
O1A—Cr1A—N4A	93.19 (8)	N3A—C6A—H6AB	110.2
N3A—Cr1A—N4A	83.74 (9)	H6AA—C6A—H6AB	108.5
N1A—Cr1A—N4A	90.77 (9)	N4A—C7A—C6A	108.8 (2)
O3A—Cr1A—N2A	92.69 (8)	N4A—C7A—H7AA	109.9
O1A—Cr1A—N2A	92.78 (8)	C6A—C7A—H7AA	109.9
N3A—Cr1A—N2A	90.86 (9)	N4A—C7A—H7AB	109.9
N1A—Cr1A—N2A	84.17 (9)	C6A—C7A—H7AB	109.9
N4A—Cr1A—N2A	171.96 (8)	H7AA—C7A—H7AB	108.3
C11A—O1A—Cr1A	114.69 (16)	N4A—C8A—C9A	113.4 (2)
C12A—O3A—Cr1A	115.17 (17)	N4A—C8A—H8AA	108.9
C10A—N1A—C1A	109.6 (2)	C9A—C8A—H8AA	108.9
C10A—N1A—Cr1A	117.10 (17)	N4A—C8A—H8AB	108.9
C1A—N1A—Cr1A	105.39 (15)	C9A—C8A—H8AB	108.9
C10A—N1A—H1A	108.1	H8AA—C8A—H8AB	107.7
C1A—N1A—H1A	108.1	C10A—C9A—C8A	114.2 (3)
Cr1A—N1A—H1A	108.1	C10A—C9A—H9AA	108.7
C3A—N2A—C2A	113.4 (2)	C8A—C9A—H9AA	108.7
C3A—N2A—Cr1A	118.47 (19)	C10A—C9A—H9AB	108.7
C2A—N2A—Cr1A	108.32 (17)	C8A—C9A—H9AB	108.7
C3A—N2A—H2A	105.2	H9AA—C9A—H9AB	107.6
C2A—N2A—H2A	105.2	N1A—C10A—C9A	112.5 (3)
Cr1A—N2A—H2A	105.2	N1A—C10A—H10A	109.1
C5A—N3A—C6A	111.0 (2)	C9A—C10A—H10A	109.1
C5A—N3A—Cr1A	117.56 (18)	N1A—C10A—H10B	109.1
C6A—N3A—Cr1A	105.70 (17)	C9A—C10A—H10B	109.1
C5A—N3A—H3A	107.4	H10A—C10A—H10B	107.8
C6A—N3A—H3A	107.4	O2A—C11A—O1A	124.3 (3)
Cr1A—N3A—H3A	107.4	O2A—C11A—C12A	121.5 (2)
C7A—N4A—C8A	112.9 (2)	O1A—C11A—C12A	114.15 (18)
C7A—N4A—Cr1A	108.49 (17)	O4A—C12A—O3A	125.2 (3)
C8A—N4A—Cr1A	117.86 (19)	O4A—C12A—C11A	121.2 (2)
C7A—N4A—H4A	105.5	O3A—C12A—C11A	113.63 (19)
C8A—N4A—H4A	105.5	O3B2—Cr2B—O4B2	118.5 (8)
Cr1A—N4A—H4A	105.5	O3B2—Cr2B—O2B2	111.1 (5)
C2A—C1A—N1A	108.4 (2)	O4B2—Cr2B—O2B2	104.7 (8)
C2A—C1A—H1AA	110.0	O2B1—Cr2B—O3B1	106.5 (6)
N1A—C1A—H1AA	110.0	O2B1—Cr2B—O4B1	106.1 (6)
C2A—C1A—H1AB	110.0	O3B1—Cr2B—O4B1	103.8 (3)
N1A—C1A—H1AB	110.0	O3B2—Cr2B—O1B	112.5 (4)
H1AA—C1A—H1AB	108.4	O4B2—Cr2B—O1B	128.2 (8)
N2A—C2A—C1A	109.2 (2)	O2B1—Cr2B—O1B	115.3 (6)
N2A—C2A—H2AA	109.8	O2B2—Cr2B—O1B	62.6 (3)
C1A—C2A—H2AA	109.8	O3B1—Cr2B—O1B	117.0 (3)
N2A—C2A—H2AB	109.8	O4B1—Cr2B—O1B	107.1 (3)
C1A—C2A—H2AB	109.8	O3B2—Cr2B—O1B^i^	109.3 (4)
H2AA—C2A—H2AB	108.3	O4B2—Cr2B—O1B^i^	107.5 (8)
N2A—C3A—C4A	113.8 (2)	O2B1—Cr2B—O1B^i^	100.0 (6)
N2A—C3A—H3AA	108.8	O2B2—Cr2B—O1B^i^	104.8 (3)
C4A—C3A—H3AA	108.8	O3B1—Cr2B—O1B^i^	85.3 (3)
N2A—C3A—H3AB	108.8	O4B1—Cr2B—O1B^i^	148.4 (2)
C4A—C3A—H3AB	108.8	O1B—Cr2B—O1B^i^	44.0 (3)
H3AA—C3A—H3AB	107.7	O1B^i^—O1B—Cr2B	74.5 (3)
C5A—C4A—C3A	114.7 (3)	O1B^i^—O1B—O2B2	128.7 (5)
C5A—C4A—H4AA	108.6	Cr2B—O1B—O2B2	57.1 (3)
C3A—C4A—H4AA	108.6	O1B^i^—O1B—Cr2B^i^	61.5 (3)
C5A—C4A—H4AB	108.6	Cr2B—O1B—Cr2B^i^	136.0 (3)
C3A—C4A—H4AB	108.6	Cr2B—O2B2—O1B	60.2 (3)
H4AA—C4A—H4AB	107.6	H1O1—O1S—H2O1	104 (2)
N3A—C5A—C4A	112.4 (3)	H1O2—O2S—H2O2	122 (3)
N3A—C5A—H5AA	109.1	H1O3—O3S—H2O3	105 (2)
C4A—C5A—H5AA	109.1	H1O4—O4S—H2O4	114 (3)
			
C10A—N1A—C1A—C2A	174.0 (2)	O2A—C11A—C12A—O4A	−1.2 (4)
Cr1A—N1A—C1A—C2A	47.1 (2)	O1A—C11A—C12A—O4A	178.8 (2)
C3A—N2A—C2A—C1A	166.9 (3)	O2A—C11A—C12A—O3A	178.4 (2)
Cr1A—N2A—C2A—C1A	33.3 (3)	O1A—C11A—C12A—O3A	−1.6 (3)
N1A—C1A—C2A—N2A	−54.5 (3)	O3B2—Cr2B—O1B—O1B^i^	−95.0 (7)
C2A—N2A—C3A—C4A	−75.2 (3)	O4B2—Cr2B—O1B—O1B^i^	74.6 (12)
Cr1A—N2A—C3A—C4A	53.4 (3)	O2B1—Cr2B—O1B—O1B^i^	77.7 (9)
N2A—C3A—C4A—C5A	−66.3 (4)	O2B2—Cr2B—O1B—O1B^i^	162.1 (8)
C6A—N3A—C5A—C4A	177.8 (2)	O3B1—Cr2B—O1B—O1B^i^	−48.7 (7)
Cr1A—N3A—C5A—C4A	−60.4 (3)	O4B1—Cr2B—O1B—O1B^i^	−164.6 (5)
C3A—C4A—C5A—N3A	69.8 (4)	O3B2—Cr2B—O1B—O2B2	103.0 (6)
C5A—N3A—C6A—C7A	175.0 (2)	O4B2—Cr2B—O1B—O2B2	−87.4 (11)
Cr1A—N3A—C6A—C7A	46.5 (3)	O1B^i^—Cr2B—O1B—O2B2	−162.1 (8)
C8A—N4A—C7A—C6A	168.8 (3)	O3B2—Cr2B—O1B—Cr2B^i^	−95.0 (7)
Cr1A—N4A—C7A—C6A	36.2 (3)	O4B2—Cr2B—O1B—Cr2B^i^	74.6 (12)
N3A—C6A—C7A—N4A	−55.8 (3)	O2B1—Cr2B—O1B—Cr2B^i^	77.7 (9)
C7A—N4A—C8A—C9A	−72.7 (4)	O2B2—Cr2B—O1B—Cr2B^i^	162.1 (8)
Cr1A—N4A—C8A—C9A	55.1 (3)	O3B1—Cr2B—O1B—Cr2B^i^	−48.7 (7)
N4A—C8A—C9A—C10A	−67.0 (4)	O4B1—Cr2B—O1B—Cr2B^i^	−164.6 (5)
C1A—N1A—C10A—C9A	179.0 (2)	O1B^i^—Cr2B—O1B—Cr2B^i^	0.005 (1)
Cr1A—N1A—C10A—C9A	−61.1 (3)	O3B2—Cr2B—O2B2—O1B	−105.2 (5)
C8A—C9A—C10A—N1A	70.3 (4)	O4B2—Cr2B—O2B2—O1B	125.8 (8)
Cr1A—O1A—C11A—O2A	−178.9 (2)	O1B^i^—Cr2B—O2B2—O1B	12.8 (5)
Cr1A—O1A—C11A—C12A	1.1 (3)	O1B^i^—O1B—O2B2—Cr2B	−22.3 (10)
Cr1A—O3A—C12A—O4A	−179.2 (2)	Cr2B^i^—O1B—O2B2—Cr2B	−138.9 (13)
Cr1A—O3A—C12A—C11A	1.2 (3)		

Symmetry code: (i) −*x*+2, −*y*, −*z*+1.

### Hydrogen-bond geometry (Å, º)

**Table:**

*D*—H···*A*	*D*—H	H···*A*	*D*···*A*	*D*—H···*A*
N1*A*—H1*A*···O4*A*^ii^	0.98	1.99	2.804 (3)	139
N2*A*—H2*A*···O1*S*	0.98	2.00	2.894 (3)	150
N3*A*—H3*A*···O2*A*^ii^	0.98	1.89	2.842 (3)	163
N4*A*—H4*A*···O2*B*1^iii^	0.98	2.25	3.100 (16)	144
N4*A*—H4*A*···O4*B*1^iii^	0.98	2.37	3.108 (6)	132
N4*A*—H4*A*···O4*B*2^iii^	0.98	2.15	3.05 (2)	151
O1*S*—H1*O*1···O4*B*1	0.85 (1)	2.33 (5)	2.876 (6)	123 (5)
O1*S*—H1*O*1···O3*B*2	0.85 (1)	2.20 (4)	2.903 (10)	141 (5)
O2*S*—H2*O*2···O3*S*	0.85 (1)	1.91 (2)	2.729 (6)	164 (6)
O3*S*—H1*O*3···O3*B*1	0.85 (1)	1.93 (3)	2.725 (6)	156 (7)
O3*S*—H1*O*3···O3*B*2	0.85 (1)	2.28 (2)	3.113 (10)	167 (6)
O3*S*—H2*O*3···O1*A*^iv^	0.85 (1)	2.52 (3)	3.301 (4)	154 (6)
O3*S*—H2*O*3···O2*A*^iv^	0.85 (1)	2.21 (5)	2.911 (5)	140 (6)
O4*S*—H1*O*4···O4*A*	0.84 (1)	2.11 (4)	2.834 (4)	144 (6)
O4*S*—H2*O*4···O2*S*	0.85 (1)	1.89 (2)	2.723 (5)	166 (6)

Symmetry codes: (ii) *x*−1, *y*, *z*; (iii) *x*, −*y*+1/2, *z*+1/2; (iv) *x*, −*y*+1/2, *z*−1/2.
